# Supplementary material for: High-energy spin fluctuation in low-Tc iron-based superconductor LaFePO0.9
Source: Sci Rep. 2018 Nov 5;8:16343. doi: 10.1038/s41598-018-33878-x (PMC6218523; doi:10.1038/s41598-018-33878-x)
Supplement: Supplementary file 1 — Supplementary Information [file 41598_2018_33878_MOESM1_ESM.docx]

# Supplementary Information on High-energy spin fluctuation

**in low-***T*c **iron-based superconductor LaFePO**0.9

## Motoyuki Ishikado1,2,3,*, Shin-ichi Shamoto2,+, Katsuaki Kodama4, Ryoichi Kajimoto5, Mitsutaka Nakamura5, Tao Hong6, and Hannu Mutka7

1Neutron Science and Technology Center, Comprehensive Research Organization for Science and Society (CROSS), Tokai, Naka, Ibaraki 319-1106, Japan

2Advanced Science Research Center, Japan Atomic Energy Agency (JAEA), Tokai, Ibaraki 319-1195, Japan

3Electronics and Photonics Research Institute, National Institute of Advanced Industrial Science and Technology (AIST), Tsukuba, Ibaraki 305-8562, Japan

4Materials Sciences Research Center, Japan Atomic Energy Agency (JAEA), Tokai, Ibaraki 319-1195, Japan

5J-PARC Center, Japan Atomic Energy Agency (JAEA), Tokai, Ibaraki 319-1195, Japan

6Neutron Scattering Division, Oak Ridge National Laboratory (ORNL), Oak Ridge, Tennessee 37831, USA

7Institut Laue-Langevin (ILL), 71 avenue des Martyrs CS 20156, F - 38042 Grenoble Cedex 9, France

**Low-energy inelastic neutron scattering of LaFePO_0_*_._*_9_**

To search for the magnetic resonance mode of LaFePO_0_*_._*_9_, the spin fluctuations were studied at two temperatures, below and above *T*_c_ using IN5 and CTAX spectrometers, because the magnetic excitation can be largely enhanced in the corresponding *Q-E* regions. The energy was estimated based on the simple linear relationship between *T*_c_ and the magnetic resonance peak energy[1](#_bookmark0)0–13. In the case of LaFePO_0_*_._*_9_, the resonance energy was estimated to be about 2.4 meV due to the low-*T*_c_ value of about 5 K.

Figures S1(a) and (b) show the 2D *Q-E* plots of the dynamical structure factor *S*(*Q,E*) at *T* =1.5 and 8.0 K measured at IN5 in ILL. The incident neutron wave length was *λ* =4.5 A˚ . Note that the data measured in the empty can filled with He gas have been subtracted from the sample data to eliminate the background. To see the details, the constant-*E* cuts of the *S*(*Q,E*) were also obtained, as shown in Fig. S2.

As mentioned in the main text, line-node symmetry was proposed based on the *T* -dependence of the magnetic penetration depth and thermal conductivity measurements30, 31. According to their reports, an extended s-wave model was proposed whose node position is on the electron Fermi surface (FS) 31. If the line node appears on the electron FS, two scattering processes are expected in addition to the normal one (inter-band scattering due to the Γ-M point FS nesting) : (1) inter-band scattering between two electron FSs (M points), and (2) intra-band scattering inside a FS. Therefore, we anticipated the magnetic excitation and accompanying resonance peak of LaFePO_0_*_._*_9_ at lower- and higher-*Q* positions than the original *Q* position at ~ 1.1Å^-1^. Constant-*E* cuts in the *E*-range from 1.2 to 2.6 meV are shown in Fig. S2. There seem no substantial peaks in these cuts. Although the data points are scattered, the spin fluctuations are strongly suppressed in this *Q-E* region. As for the superconducting state, we had calculated the generalized spin susceptibility *χ*”(*E*) for various symmetry models (*i.e.* horizontal node, *s±* wave, and *d_xy_*-wave (vertical node on a hole FS) ) on the basis of the multi-orbital random phase approximation (RPA) 37. Results of the RPA calculation suggested that in the case of a vertical line node (*dxy*-wave case), the resonant enhancement ratio was < 1, namely, magnetic excitation decreased in the superconducting state. This was due to the effect of the BCS coherence factor since if the line node lies vertically, the sign-reversal scattering process between Γ-*M* FS decreases. Therefore, the magnetic resonance mode of LaFePO_0.9_ at *Q ~* 1*.*1 Å^-1^ may appear as suppressed magnetic excitation in the superconducting state. In the *Q-E* region, there are peaks around *Q*=0.9 and 1.8 Å^-1^at *E*=2.4 meV. However, these peaks are observed only at *E*=2.4 meV. Moreover, the signal intensity is very weak in comparison to the ~1.1 Å^-1^magnetic excitation peak observed in the optimally doped LaFeAsO_0_*_._*_918_F_0_*_._*_082_. To see the upper limit of the peak-like structure, we fit it with two Gaussian functions. The absolute value of *χ*”(*E*) is shown in Fig. 4, which is considerably smaller than the others. Based on these results, we conclude that there are no spin fluctuations in this *Q-E* range. This result is consistent with a previous report25.

1

## References

**37.** Ishikado M. *et al. s*±-like spin resonance in the iron-based nodal superconductor BaFe_2_(As_0_*_._*_65_P_0_*_._*_35_)_2_ observed using inelastic neutron scattering. *Phys. Rev. B* **84**, 144517 (2011).


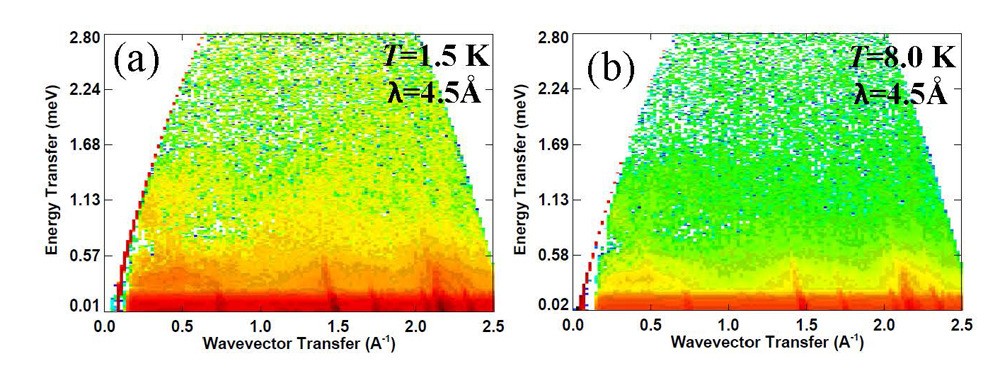


**Figure S1.** Plots of 2D *S*(*Q,E*) for LaFePO_0.9_ at (a) *T* =1.5 K and (b) *T* =8.0 K with incident neutron wave length *λ* =4.5A˚ .

**Figure S2.** Constant-*E* cuts of *S*(*Q,E*) plots in Fig. S1 in the *E*-range from 1.2 to 2.6 meV, measured at (a) *T* =1.5 K and (b) *T* =8.0 K. (c) The differences of the dynamical structure factor *S*(*Q,E*) between *T* = 1.5 and 8.0 K. The scatter patterns are vertically shifted for clarity. Broken lines are guides to the eye.
